# Spermatozoa Induce Maternal Mononuclear Cells for Production of Antibody with Cytotoxic Activity on Paternal Blood Mononuclear Cells

**DOI:** 10.22074/cellj.2021.7157

**Published:** 2021-07-17

**Authors:** Nasrin Sereshki, Alireza Andalib, Mohadeseh Toghyani, Hossein Motedayyen, Mohammad Sadegh Hesamian, Abbas Rezaei, David Wilkinson

**Affiliations:** 1. Department of Immunology, School of Medicine, Isfahan University of Medical Sciences, Isfahan, Iran; 2. Asadabad School of Medical Science, Asadabad, Iran; 3. Autoimmune Diseases Research Centre, Kashan University of Medical Sciences, Kashan, Iran; 4. University of Aberdeen, Scotland, UK

**Keywords:** Antibodies, Antigen, Cell Cytotoxicity, Spermatozoa

## Abstract

**Objective:**

The maternal immune response to paternal antigens is induced at insemination. We believe that pregnancy protective alloantibodies, such as anti-paternal cytotoxic antibody (APCA), may be produced against the paternal antigens in the context of stimulated immunity at insemination and that they increase during pregnancy. APCA is necessary for pregnancy. It is directed towards paternal human leucocyte antigens (HLAs) and has cytotoxic activity against paternal leucocytes. The present study aims to determine whether APCA is produced by the maternal peripheral blood mononuclear cells (PBMCs) in contact with the husband’s spermatozoa and to evaluate the relation of APCA production with HLA class I and II expressions by spermatozoa in fertile couples.

**Materials and Methods:**

This cross-sectional study included 30 fertile couples with at least one child. The maternal PBMCs were co-cultured with the husband’s spermatozoa and the supernatant was assessed for the presence of IgG by ELISA. Cytotoxic activity of the supernatant on the husband’s PBMCs was assessed by the complement-dependent cytotoxicity (CDC) assay.

**Results:**

IgG was produced in all co-cultures, and the mean level of supernatant IgG was 669 ng/ml. The cytotoxic activity of the supernatant was observed in all the supernatant obtained from the co-cultures. The mean percentage of APCA in supernatant was 73.93%.

**Conclusion:**

Based on the results of this study it can be concluded that APCA may be a natural anti-sperm antibody (ASA), which can be produced during exposure to spermatozoa and may have some influence before pregnancy. Further research is required to determine the role of APCA before pregnancy.

## Introduction

Studies suggest that the maternal immune response to paternal antigens is induced prior to conception and possibly during insemination. After sexual intercourse, the infiltration of neutrophils, macrophages and lymphocytes in the female reproductive tract (FRT) due to the presence of immune stimulating factors in semen has been clearly shown. The consequence of this inflammation is the adaptation of maternal-innate and adaptive immune responses for the occurrence of pregnancy (1-3). In immunity against semen, the immune effector response and the regulatory response (regulatory T cells or Tregs) are induced. Evidence suggests a delicate balance between the effector and regulatory responses, and this may be required for pregnancy to occur. One of the roles of the immune effector response could be preparation of tissue destruction factors such as metalloproteinase enzymes and inflammatory cytokines needed for embryo implantation. A role of the regulatory response is prevention of an excessive effector response (4). Disruptions in balance between the effector and regulatory response results in pregnancy aberrations, such as recurrent spontaneous abortion (RSA) (5). These findings suggest that induction of both effector and regulatory responses against semen antigens is essential for pregnancy occurrence.

Despite many studies on the induction of the maternal immune response to semen, we could not find any study that has addressed beneficial humoral immunity (antibody production) against semen or spermatozoa. We believe that pregnancy-protective alloantibodies against the paternal antigen may be produced in the context of stimulated immunity at insemination and they increase in pregnancy due to the increase of paternally-derived foetal antigens in the maternal blood circulation. Therefore, any destruction of semen or spermatozoa antigenicity may result in disturbed production of alloantibodies and pregnancy complications. This supposition, the production of pregnancy-protective alloantibody at insemination, was presented when we observed that absence of pregnancy-protective alloantibodies resulted in RSA (6, 7). Thus, pregnancy-protective alloantibodies must be present before pregnancy and are necessary for pregnancy occurrence; otherwise, RSA occurs. 

Anti-paternal cytotoxic antibody (APCA) is one of the pregnancy alloantibodies which belongs to the IgG class and is directed to paternal human leucocyte antigens (HLAs) (8). There is scant information about its function and mechanism of action. The absence of APCA is related to RSA. There is a relationship between APCA development after lymphocyte therapy and the success of this treatment in improving live birth rates in RSA women (6, 9). This suggests that APCA may be produced upon contact with HLAs in semen and spermatozoa. Although there is considerable controversy surrounding HLA class I and II expression by spermatozoa (10-19), we recently demonstrated that these antigens were expressed by spermatozoa (20). Given this description, it is very likely that APCA would be produced in contact with spermatozoa in the context of stimulated immunity at insemination and help pregnancy to occur. We hypothesize that APCA production in contact with spermatozoa may benefit humoral immunity following insemination.

As mentioned, we have not found any study that assessed beneficial humoral immunity against spermatozoa. Thus, to commence the study about maternal humoral immunity against spermatozoa, we aim to determine: i. Whether antibody is produced by the female’s peripheral blood mononuclear cells (PBMCs) in the presence of the husband’s spermatozoa, ii. Whether APCA is produced by the female’s PBMCs in contact with the husband’s spermatozoa, and iii. The correlation of APCA and antibody production with HLA class I and II expressions by spermatozoa. To the best of our knowledge, no study has addressed these topics. 

## Materials and Methods

### Subjects

In this cross-sectional study, we included 30 fertile couples aged 28-41 years who had at least one child. The anti-sperm antibody (ASA) test was negative in these couples. The maternal participants denied any history of pregnancy complications (e.g., ectopic pregnancy, preterm and post-term labour or preeclampsia), blood transfusion or organ transplantation. The husband of each woman had normal semen status according to the criteria from 2010 guidelines of the World Health Organization. None of the male partners had any history of genital tract disorders such as infections, undescended testis, inguinoscrotal surgery, genital trauma or testicular torsion. 

Five women, aged 28-42 years, who were virgins and had no history of semen contact, blood transfusion or organ transplantation were recruited to this study as the uncontacted negative control. Because they had no history of alloantigen contact, we expected that PBMCs obtained from these women would not produce antibodies in the presence of spermatozoa. We chose an age and sex matched control to remove the effect of age and sex related confounding factors such as hormonal factors, variation of microbiota with age and other factors that have important effects on the immune system. More reliable results could be acquired when the only difference between virgin women (uncontacted control) and the maternal women (with partners) was the lack of semen contact. 

Informed consent was obtained from all subjects. The Ethics Committee of Isfahan University of Medical Sciences (Isfahan, Iran) approved this protocol (approval letter: IR.MUI.REC.1395.3.480). 

### Purification of spermatozoa

Semen samples were collected by masturbation after 2-3 days of sexual abstinence. Sampling was performed under sterile conditions to prevent false results due to a change of spermatozoa antigenicity induced by toll-like receptors on the spermatozoa in response to microbial antigens. After liquefaction, semen quality was assessed according to WHO standard guidelines (WHO, 2010) and couples were excluded if the husband had an abnormal semen quality. We added 2 ml of AllGrad Wash (LifeGlobal® Group, Canada) to the liquefied semen sample, and centrifuged the sample at 350 g for 10 minutes. The pellet was re-suspended in 1 ml of AllGrad Wash® and 2 gradient solutions (95% and 45%) prepared from AllGrad 100% (LifeGlobal® Group, Canada). In each tube, 1 ml of AllGrad 90% gradient, followed by 1 ml of AllGrad 45% gradient and then 1 ml of the spermatozoa suspension were carefully layered. The tubes were centrifuged at 400 g for 18 minutes. The spermatozoa pellet at the bottom of the centrifuge tubes was washed with AllGrad Wash® and then re-suspended in Ham’s F-10 medium (Dacell, Iran) with 1% bovine serum albumin (CMG, Iran). Ham’s F-10 medium was used because of its antioxidant properties, which makes it a suitable medium for spermatozoa (21).

### Flow cytometry assay 

We added 1×10^6^ spermatozoa per 100 µl medium was added to two tubes of each purified spermatozoa sample. One tube was incubated with phycoerythrin (PE) mouse anti-human HLA-ABC (clone: G46-2.6, BD Pharmingen, USA) and the other was incubated with PE mouse anti-human HLA-DR (clone: G46-6, BD Pharmingen, USA) at room temperature for 30 minutes. After two washes with AllGrad Wash® (400 g for 5 minutes), the spermatozoa were run through a flow cytometer (BD FACSCalibur, USA). Data from at least 100000 events were collected using forward scatter (FSC) and side angle of light scatter (SSC), a logarithmic amplifier. Fluorescence data were obtained with the logarithmic amplifier. To determine background fluorescence (auto-fluorescence and non-specific binding of antibodies), we used two isotype controls (mouse IgG1, κ [clone: G46-2.6, BD Pharmingen, USA] and mouse IgG2a, κ [clone: G46-6, BD Pharmingen, USA]). An antibody titration was performed and we selected the optimal titre that displayed the minimum background to eliminate any background fluorescence. FSC versus SSC gating was used to identify spermatozoa and remove debris. To determine the percentage of HLA class I and II positive spermatozoa, SSC versus logarithmic FL2 (PE) gating was used. The cell viability test was not performed because abnormal and dead spermatozoa were removed by AllGrad solution before staining. The data were analysed using FlowJo version10 software.

### Isolation of peripheral blood mononuclear cells and performing co-culture

After taking 5 ml of heparinized venous blood, the PBMCs were separated by centrifugation on a Ficoll-Hypaque (Lymphoprep, Sigma, USA) density gradient. Cells at the interface were harvested, washed twice and suspended in complete RPMI 1640 medium supplemented with HEPES, L-glutamine, penicillin (100 U/ml), streptomycin (10 mg/ ml), 2-mercaptoethanol (2×10^-5^ M) and 20% autologous serum. In this suspension, we adjusted the cell concentration to 1×10^6^ cells/ml. A 2 ml suspension (2×10^6^ PBMCs) was transferred to 24-well plates and cultured in the presence of 5×10^6^ spermatozoa. As the negative control, 2×10^6^ maternal PBMCs without spermatozoa were cultured in parallel to the co-cultures. Cells were then incubated at 37˚C in a humidified 5% CO_2_ atmosphere. After four days, cells were washed three times and the pellet was re-suspended in 2 ml complete RPMI 1640 medium in which autologous serum was replaced by 20% foetal calf serum (FCS). After incubation for eight days at 37˚C in a humidified 5% CO_2_ atmosphere, the supernatants were harvested, aliquoted and kept at -80°C for future use.

For the uncontacted negative control, blood was taken from the women volunteers who were virgins. The PBMCs were separated and these cells were co-cultured with the pooled spermatozoa obtained from five normal donors. The culture procedure was similar to that mentioned above. There were two controls: i. Negative control (culture of PBMC alone from the maternal women) and ii. Uncontacted negative control (co-culture PMBC from virgin women with pooled spermatozoa). The first control was run to ensure that any unknown factors did not lead to the production of antibodies, and that the production of antibodies in the co-cultures was because of the presence of spermatozoa. The second control was performed to ensure that antibodies produced in the presence of spermatozoa indicated that sensitization had previously occurred. In other words, the uncontacted negative control was run to confirm that the female humoral immune response (antibody production) was induced by spermatozoa at the time of insemination. 

### Enzyme-linked immunosorbent assay analysis

The concentration of IgG in the supernatant was measured using an enzyme-linked immunosorbent assay (ELISA) kit in accordance with the manufacturer’s protocol [IgG (Total) Human Uncoated ELISA Kit, Invitrogen, USA]. Normal serum samples that contained IgG were used as the positive control.

### Complement-dependent cytotoxicity assay for determining the anti-paternal cytotoxic antibody titre in the supernatants

We assessed the APCA percentage by cross-matching between supernatants (1:64 dilution) and freshly prepared paternal PBMCs. The test was performed in triplicate in Terasaki plates covered with light paraffin oil. We mixed one µl of paternal PBMCs suspension (density: 2×10^3^ cells/ml) with 1 µl supernatant. For quality control of the complement-dependent cytotoxicity (CDC) assay and to ensure accuracy of the assay, two control samples – a negative control (antibody without cytotoxic antibody) and positive control (antibody with cytotoxic activity) were used. The positive control was the serum of the women at the third trimester of pregnancy because it contained a high level of APCA. APCA increases to detectable levels from the 28^th^ week of pregnancy until four weeks after delivery, after which it decreases to an undetectable level (22). The serum of these women was mixed with their male partner’s PBMCs. The negative control consisted of supernatants obtained from co-culturing PBMCs from virgin women with pooled spermatozoa, which lacked any antibodies. After one hour at room temperature, we added 5 µl of rabbit complement (Inno-Train, Germany). One µl eosin dye was added to the wells after one hour of incubation at room temperature, followed by 5 µl of formalin (37%). The test plates were left overnight to allow the cells to settle. The plates were read using a phase contrast microscope (Olympus, Japan). The number of dead cells among 1000 PBMCs was determined and reported as the percentage of APCA.

### Statistical analysis

Descriptive analysis of the percentage of HLA class I and II expression by spermatozoa, IgG concentration and percentage of APCA in supernatant included the mean and standard deviation (SD). A Pearson product-moment correlation coefficient was used to assess the relationship between variables. All data analysis was performed using IBM SPSS statistics 25 software. A P≤0.05 was considered statistically significant.

## Results

### Evaluation of human leucocyte antigen class I and II on the surface of spermatozoa

**Fig.1:**
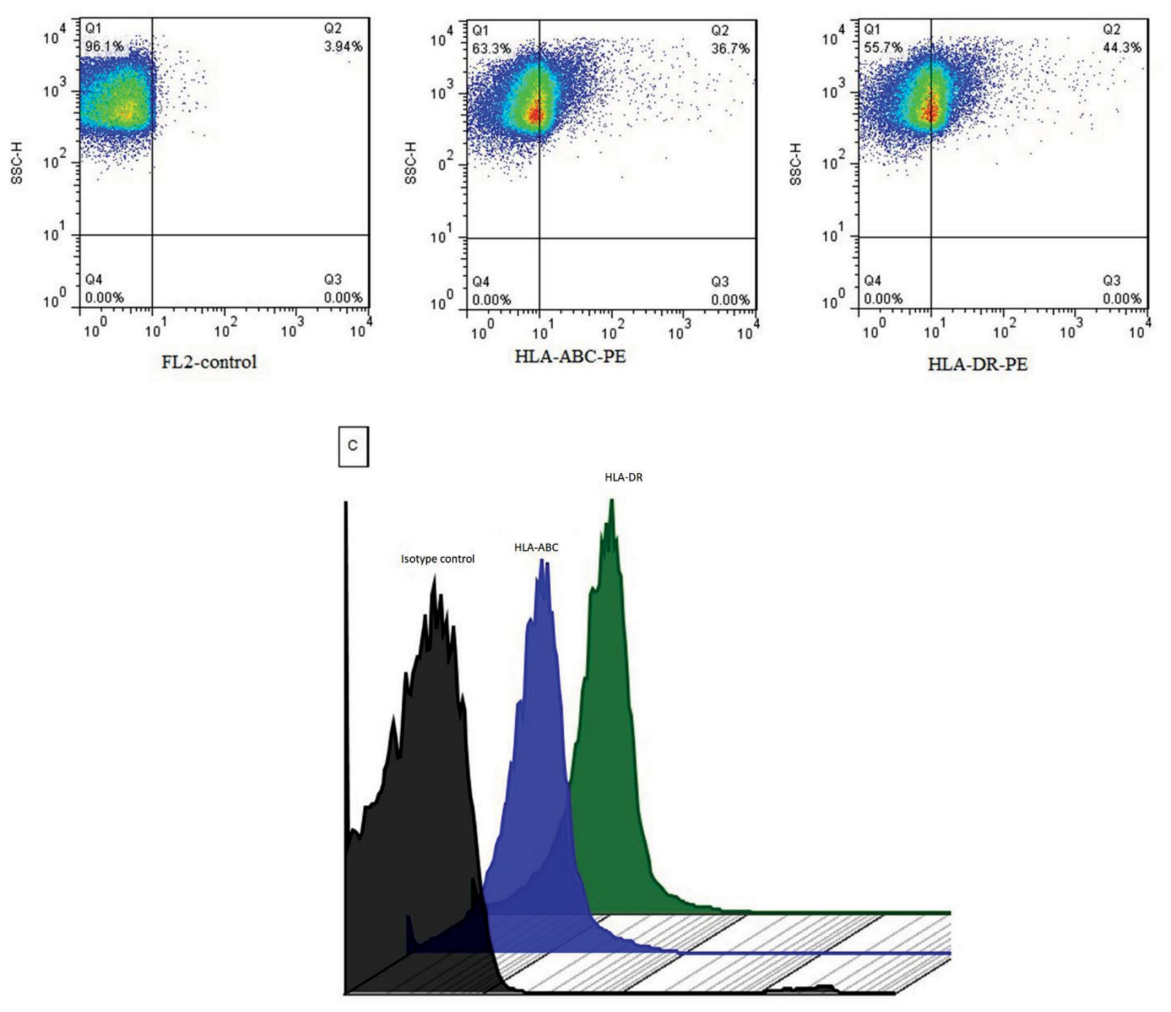
Representative of the flow cytometry dot plot and histogram overlay. Purified spermatozoa were assessed for expression of human leucocyte antigen (HLA) class I and II by flow cytometry. The dot plot is used to show the percentage of HLA class I and HLA class II positive spermatozoa. C is a representative stagger histogram overlay of the isotype control with the experimental sets to show the background level after minimizing background auto-fluorescence.

Flow cytometric results showed that 25.77 ± 9.9% of spermatozoa expressed HLA class I on their surface and that 29.95 ± 10.10% of spermatozoa expressed HLA class II. Figure 1 presents a representative flow cytometry dot plot and histogram overlay. 

### IgG concentration in the supernatant

Supernatant IgG levels were measured by ELISA. IgG was detected in all of the supernatants. The mean ± SD of the IgG concentration in supernatants was 669 ± 132 ng/ml. The IgG concentration in the negative control was 3.20 ± 2.31 ng/ml and in the uncontacted negative control, it was 3.2 ± 2.90 ng/ml. 

### Percentage of anti-paternal cytotoxic antibody in the supernatant

**Fig.2:**
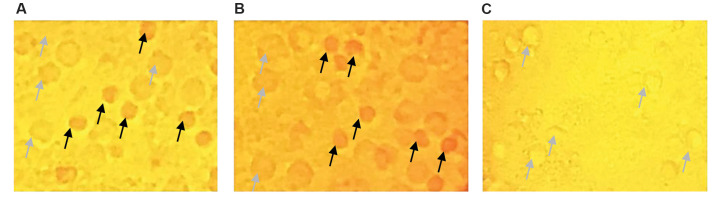
Phase contrast microscope images of the husbands’ peripheral blood mononuclear cells (PBMCs) after the complement-dependent cytotoxicity (CDC) assay. Eosin dye was taken up by the dead cells and they became a dark colour. **A. **Positive control, **B.** Test (cytotoxic activity of supernatant on paternal PBMCs, and **C. **Negative control. Black arrow; Dead cells and Gray arrow; Live cells.

CDC results showed that all supernatants were positive for APCA. The percentage of APCA in the supernatant was 73.93 ± 16.01%. The percentage of dead cells in the negative controls was 3.12 ± 2.96%. Figure 2 shows phase contrast microscope images of PBMCs after the CDC assay.

### Correlation assessments

A Pearson product-moment correlation coefficient was computed to assess the relationship between APCA production and HLA class I and II expression by spermatozoa. There was a positive correlation between these factors [P=0.011 (HLA-I) and P=0.013 (HLA-II), Table 1].

**Table 1:** Correlation results


Pearson correlation		IgG (ng/ml)	APCA (%)

HLA-I %	r	0.459^*^	0.509^*^^*^
	P value	0.011	0.004
	n	30	30
HLA-II %	r	0.449^*^	0.499^*^^*^
	P value	0.013	0.005
	n	30	30


*; Correlation is significant at the 0.05 level (2-tailed), **; Correlation is significant at the 0.01 level (2-tailed), n; Number; HLA; Human leucocyte antigens, APCA; Anti-paternal cytotoxic antibody, and r; Correlation coefficient.

## Discussion

Despite numerous studies about the cellular immunity that is induced in FRT at insemination, the humoral immunity and the production of useful antibodies against spermatozoa (ASA) has not been assessed. In this study, we show, for the first time, that IgG and APCA are produced by the wife’s PBMCs in the presence of the husband’s spermatozoa. Furthermore, we show a positive correlation between HLA expression by spermatozoa and antibody production. To the best of our knowledge, this is the first study that has investigated the ability of spermatozoa to induce the production of IgG and APCA. We could not find any study that investigated the correlation between HLA expression on spermatozoa and the induction of immunity (e.g., antibody production) by spermatozoa.

The production of antibodies (IgG) against an antigen in vitro by PBMCs is indicative of the stimulation of the immune response against the antigen and development of memory B lymphocytes that respond to the presence of the antigen in the body. In this study, we have shown that the antibody (IgG) was produced ASA when the wife’s PBMCs were co-cultured with the husband’s spermatozoa. This result suggested development of memory humoral immunity ASA in the wife (or maternal) body. 

Regarding these produced antibodies, the question arises as to which antigens of spermatozoa have the capability to cross-react with paternal lymphocyte antigens. One of the most likely antigens are the HLA molecules. This study showed a positive correlation between HLA class I and II and APCA production. It is possible that APCA, which is originally developed through exposure to paternally derived foetal antigens during pregnancy, can cross-react with the same antigens on spermatozoa. However, it should be noted that the immune system is exposed to semen antigen before foetal antigen. 

Our results show that fertile women produce antibodies against spermatozoa (ASA). In contrast to our results, many studies have shown that ASA in serum or in FRT secretions results in infertility. Also, numerous studies have sought to determine the antigen specificity of ASA. Despite the large number of studies on the pathogenicity of ASA for pregnancy, the presence of this antibody was reported in a very small percentage of fertile women. Accordingly, the results of our study showed that fertile women have immunological memory of antibody production in the presence of the husband’s spermatozoa (23-25). However, the question is raised as to why APCA, a type of ASA, is not detectable in most normal parous women. Two possible reasons can be suggested. First, under normal conditions, APCA producing plasma cells migrate in the FRT and locally produce a small amount of antibody so that the release of antibody into the blood is very limited, and often not identifiable by current methods. Second, APCA may be produced when first encountering spermatozoa, after which it may bind to spermatozoa and be eliminated by cells in the FRT. Consequently, the amount of unbound antibody is very low and cannot be detected in serum and cervical secretion or blood.

The question arises as to why APCA with cytotoxic activity does not lead to infertility. We believe that, in addition to not leading to infertility, APCA has some major roles for supporting pregnancy. These possible roles for APCA before pregnancy include: 

i. Opsonizing senescent and damaged spermatozoa for phagocytosis, ii. Assisting in promoting a proper immune response required for implantation and tolerance induction, iii. Because the cytotoxic activity of immune cells (Natural killer cells and cytotoxic T lymphocytes) is essential for implantation (26-28), it is probable that APCA also has a role in this cytotoxicity process. This role of APCA may occur through the antibody-dependent cell-mediated cytotoxicity (ADCC) that is intermediated by NK cells. 

Therefore, it seems logical that the APCA in normal women (which we named natural ASA) has a different function and features compared to the pathogenic ASA in infertile women. We believe that natural and pathogenic ASA may have different properties, such as antigenic specificity, glycosylation type and isotype. Further studies should be performed to assess this supposition.

Determination of the level of APCA in the serum by the CDC assay is a diagnostic test for RSA. However, the sensitivity of this method is low because of the decreased level of APCA in the serum. We intend to use APCA production in the presence of spermatozoa as a diagnostic test for RSA. To achieve this purpose, a study should be performed on a large number of fertile and RSA couples to determine a cut-off value and the normality of APCA.

## Conclusion

Based on the results of this study it can be concluded that APCA can be a natural ASA, which might be produced during exposure to spermatozoa and might have some influence before pregnancy. Further research is required to determine the role of APCA before pregnancy. 
